# Protozoal giant viruses: agents potentially infectious to humans and animals

**DOI:** 10.1007/s11262-019-01684-w

**Published:** 2019-07-09

**Authors:** Beata Tokarz-Deptuła, Paulina Niedźwiedzka-Rystwej, Paulina Czupryńska, Wiesław Deptuła

**Affiliations:** 1 Department of Immunology, Faculty of Biology, University of Szczecin, Szczecin, Poland; 2 Department of Microbiology, Faculty of Biology, University of Szczecin, Szczecin, Poland; 3 Veterinary Centre, Nicolaus Copernicus Univeristy in Toruń, Torun, Poland

**Keywords:** Protozoa, Giant virus, Virophage

## Abstract

The discovery of giant viruses has revolutionised the knowledge on viruses and transformed the idea of three domains of life. Here, we discuss the known protozoal giant viruses and their potential to infect also humans and animals.

## Introduction

Giant viruses (megaviruses) infecting protozoa (amoebae and flagellates) are microorganisms classified into NCLDVs (Nucleocytoplasmic Large DNA Viruses) [1]. The genetic material of giant viruses is typically linear but may also be in the form of circular dsDNA and is characterised by large virion sizes (above 200 nm). These viruses are also relatively independent of their host, although they usually replicate in their cytoplasm, in the “viral particle factory” [1, 2]. Their molecule has been found to have, apart from the nucleic acid (dsDNA), structures typical of organisms other than prokaryotes, inter alia the factors necessary for translation initiation or the typical transposable genome elements. These findings would suggest that giant viruses have common ancestry [2–4]. More importantly, megaviruses in protozoa are “hosts” to virophages [5–9] and have a viral defence system named MIMIVIRE (Mimivirus virophage resistant element) which has so far not been described in viruses, including those classified into NCLDVs [9–11]. Because of these facts and of the impossibility of researching the viruses on the basis of their rRNA sequences, they cannot be regarded as falling within the currently used definition of three domains. In addition, based on the analysis of sequences of the gene encoding the DNA-dependent RNA polymerase [12] that is usually performed as part of genetic characterisation of viruses, megaviruses have been classified into a group of factors standing out from the three domain classification (TRUC—things resisting uncompleted classification) [12, 13]. The TRUC group has been proposed to be a fourth domain of life, Megavirales [2, 4]. Irrespective of those data, megaviruses are classified (Tables 1, 2, 3), as mentioned above, into NCLDVs which also include viruses of the family *Asfarviridae,* infecting vertebrates, viruses of the family *Poxviridae*, infecting vertebrates and insects and viruses of the family *Iridoviridae*, infecting insects, fish and invertebrates from aquatic environments [1, 2, 14–16], as well as giant viruses of the family *Phycodnaviridae*, infecting marine algae [1, 16–18]. It should also be added that, on account of the features of megaviruses infecting amoebae and flagellates living in aquatic environments (water, waste water, fountains), permafrost (ice), the air (aerosols, air conditioners) and in the soil, and of those infecting amoebae living in sponges, corals, molluscus (mussels), insects as well as in mammals (humans, rodents, livestock—sheep, cattle), giant viruses are perceived differently, if only for their habitats, and their phylogeny is thus perceived differently, too (Tables 1, 2, 3 and [1, 2, 14–31]). Like most viruses, megaviruses are isolated by culture method, although their cultures are based on protozoa (mostly amoebae) which are infected with a sample expected to potentially contain megaviruses. In such cultures, lysis of amoebal cells in the form of plaques [32] is observed. Next, by using different amoebal species, one creates an opportunity to isolate viruses specific to a given unicellular organism and then, in order to assign them to a specific taxonomic group, they are investigated using genetic techniques, especially the sequencing, as well as visualisation by atomic force microscopy [32] and flow cytometry [33]. The database of the National Center for Biotechnology Information (NCBI) [1] currently contains over 200 giant viruses and (or) sequences infecting protozoa. For this kind of viruses, families *Mimiviridae* (Table 1) and *Marseilleviridae* (Table 2), and a group of viruses without any designated family name (Table 3) have been created.

**Table 1:** Protozoal giant viruses—family *Mimiviridae*

No.	Genera of virus	Species [ref]	Genetic material	Host	Place and year of isolation	Line/group	Virophage
1.	*Mimivirus*	Mimivirus -Acanthamoeba polyphaga mimivirus (APMV) [16]	Linear dsDNA	*Acanthamoeba (A.) polyphaga*	Cooling tower water, hospital, Bradford (Great Britain)—2003	A	Sputnik
		Mamavirus – Acanthamoeba castellani mamavirus (ACMV) [8, 61]	Linear dsDNA	*A. castellani* *A. polyphaga*	Cooling tower water, hospital, Paris (France)—2008 Soil (France)—2012		Sputnik Sputnik 3
		Lentille virus [7]	dsDNA (arrangement non-defined)	*A. polyphaga*	Contact lenses solution (France)—2012		Sputnik 2
		Samba virus [6]	dsDNA (arrangement non-defined)	*A. castellani*	Negro River Amazonia (Brasil)—2014		Rio Negro
		Kroon virus [54]	dsDNA (arrangement non-defined)	*Acanthamoeba spp*	Municipal lake (Brasil)—2015		No
		Amazonia virus [54]	dsDNA (arrangement non-defined)	*Acanthamoeba spp*	Amazonia’s river (Brasil)—2015		No
		Oyster virus [54]	dsDNA (arrangement non-defined)	*Acanthamoeba spp*	Oyster farm (Brasil)—2015		No
		Niemeyer virus [55]	dsDNA (arrangement non-defined)	*Acanthamoeba spp*	Municipal lake (Brasil)—2015		No
		Mimivirus bombay [56]	dsDNA (arrangement non-defined)	*A. castellani*	Sewage Mumbai (India)—2016		No
		Moumouvirus—Acanthamoeba polyphaga moumouvirus [42]	dsDNA (arrangement non-defined)	*A. polyphaga*	Cooling tower water (France)—2008	B	No
		Moumouvirus monve [42]	dsDNA (arrangement non-defined)	*Acanthamoeba spp*	Cooling tower water (France)—2008		No
		Saudi moumouvirus [91]	dsDNA (arrangement non-defined)	*A.polyphaga*	Hospital’s sewage in Jeddah (Saudi Arabia)—2016		No
		Courdo11 [58]	dsDNA (arrangement non-defined)	*Acanthamoeba spp*	River Le Peyron (France)—2010	C	No
		*Megavirus chilensis* [42]	dsDNA (arrangement non-defined)	*Acanthamoeba spp*	Sea water (Chile)—2011		No
		Mont1 [60]	dsDNA (arrangement non-defined)	*A. polyphaga*	Soil (Tunisia)—2014		Zamilon
		Shan virus [62]	dsDNA (arrangement non-defined)	*Acanthamoeba spp*	Human’s faeces (Tunisia)—2013		No
		LBA111 [15, 62]	dsDNA (arrangement non-defined)	*Acanthamoeba spp*	Human’s bronchoscopy specimen (Tunisia)—2013		No
2.	*Cafeteriavirus*	*Cafeteria roenbergensis virus (*CroV) [5, 65]	dsDNA (arrangement non-defined)	*Cafeteria roenbergensis*	Sea water (USA)—2011	Non-defined	Mavirus
3.	Non-defined*	Non-defined* [39]	dsDNA (arrangement non-defined)	Non-defined	Lake in Antarctica—2013	Non-defined	ALM
		Non-defined* [92]	dsDNA (arrangement non-defined)		Sheep’s rumen (USA)—2015		RVP
4.	*Klosneuvirus*	Klosneuvirus [67, 68]	dsDNA (arrangement non-defined)	*Acanthamoeba* spp.	Sewage treatment plant, Austria—2017	Non-defined	No
		Catovirus [67, 68]	dsDNA (arrangement non-defined)				
		Hokovirus [67, 68]	dsDNA (arrangement non-defined)				
		Indivirus [67, 68]	dsDNA (arrangement non-defined)				
5.	*Tupanvirus*	Tupanvirus from salt lake [67]	dsDNA (arrangement non-defined)	*A. castellanii Vermamoeba vermiformis*	Salt lake Nhecolândia (Brasil)—2018	Non-defined	No
		Tupanvirus from the ocean [67]	dsDNA (arrangement non-defined)		Ocean’s waters Campos dos Goytacazes (Brasil)—2018		

*Probably the *Mimiviridae* family members

**Table 2:** Protozoal giant viruses—family *Marseilleviridae*

No.	Genera of virus	Species [ref]	Genetic material	Host	Place and year of isolation	Line/group
1.	*Marseillevirus*	*Marseillevirus marseillevirus* (APMaV) [34, 69]	Circular dsDNA	*A. polyphaga*	Cooling tower water, Paris (France)—2007	A
		Cannes 8 virus [71]	Circular dsDNA	*Acanthamoeba spp*	Cooling tower water, Cannes (France)—2013	
		Senegalvirus [14, 66]	dsDNA (arrangement non-defined)	*Acanthamoeba* spp.	Faeces of a healthy human (Senegal)—2013	
		Melbournevirus [93]	dsDNA (arrangement non-defined)	*Acanthamoeba spp*	Pond water Melbourne (Australia)—2014	
		*Lausannevirus* [73]	Linear or circular dsDNA	*A.castellani*	River Seine (France)—2011	B
		Insectomime virus [76]	dsDNA (arrangement non-defined)	*Acanthamoeba spp*	*Eristalis tenax*—2013	C
		*Tunisia virus* [75]	dsDNA (arrangement non-defined)	*Acanthamoeba spp*	Fountain water (Tunisia)—2014	
		Brazilian *marseillevirus* [77]	dsDNA (arrangement non-defined)	*Acanthamoeba spp*	Sewage treatment plant water (Brasil)—2016	D
		Tokyovirus [78]	dsDNA (arrangement non-defined)	*Acanthamoeba spp*	Mud from Arakawa river, Tokyo (Japan)—2016	E
		Golden *marseillevirus* [79]	Circular dsDNA	*Acanthamoeba polyphaga*	Limnoperna fortunei living in river (Brasil)—2016	Non-defined
		*Marseillevirus lymphoma* [20]	dsDNA (arrangement non-defined)	*Acanthamoeba spp*	Lymph node specimen from a patent with Hodgkin’s lymphoma (France)—2016	Non-defined

No virophages defined

**Table 3:** Protozoal giant viruses—no family assigned

No.	Genera of virus	Species [ref]	Genetic material	Host	Place and year of isolation	Line/group
1.	*Pandoravirus*	*Pandoravirus salinus* [13, 14, 80, 94]	dsDNA (arrangement non-defined)	*Acanthamoeba* spp.	Deposit of river Chile (Chile)—2013	Non-defined
		*Pandoravirus dulcis* [13, 14, 80, 94]	dsDNA (arrangement non-defined)		Mud from the pond near Melbourne (Australia)—2013	
		*Pandoravirus inopinatum* [66]	dsDNA (arrangement non-defined)		Conjunctiva of a patent with keratitis—2015	
2.	*Pithovirus*	*Pithovirus sibericum* [35]	dsDNA (arrangement non-defined)	*Acanthamoeba* spp.	permafrost Siberia (Russia)—2014	
		*Pithovirus massiliensis* [84]	dsDNA (arrangement non-defined)		Waste water La Ciotat (France)—2015	
		*Cedratvirus* [81]	dsDNA (arrangement non-defined)		Sea water (Algeria)—2015	
3.	*Faustovirus*	Faustovirus E9 [85, 86]	dsDNA (arrangement non-defined)	*Vermamoeba vermiformis*	Waste water Marseille (France)—2015	E9
		Faustovirus E12 [85, 86]	Circular dsDNA			M
		Faustovirus E23 [85, 86]	dsDNA (arrangement non-defined)			M
		Faustovirus E24 [85, 86]	dsDNA (arrangement non-defined)			M
		Faustovirus D5a [86]	dsDNA (arrangement non-defined)		Waste water near Dakar (Senegal)—2015	D
		Faustovirus D3 [85, 86]	dsDNA (arrangement non-defined)			D
		Faustovirus D5b [85, 86]	dsDNA (arrangement non-defined)			D
		Faustovirus D6 [85, 86]	dsDNA (arrangement non-defined)			D
		Faustovirus Liban [85, 86]	dsDNA (arrangement non-defined)		Waste water Tripoli El Mina (Lebanon)—2015	L
4.	*Mollivirus*	*Mollivirus sibericum* [71, 88]	dsDNA (arrangement non-defined)	*Acanthamoeba castellanii*	Permafrost Siberia (Russia)—2014	Non-defined
5.	No name	*Kaumoebavirus* [57]	Circular dsDNA	*Vermamoeba vermiformis*	Waste water Jeddah (Audi Arabia)—2016	Non-defined

No virophages defined

### Giant viruses of the family *Mimiviridae*

Among the 26 megavirus species from the family *Mimiviridae* that have been described so far, 17 species make up the genus Mimivirus, 1 species makes up the genus Cafeteriavirus, 2 isolates have no species or genus name, 4 species/isolates constitute the genus Klosneuvirus, while 2 other species/isolates are members of the genus Tupanvirus (Table 1). On the basis of a phylogenetic analysis involving repeated alignment of DNA polymerase sequences, these megaviruses have been divided—but only within the genus Mimivirus—into three lines/groups (A, B and C), and 8 of them have been proven to be “hosts” to virophages (Table 1).

The first giant virus from this family (Table 1), infecting protozoa, is APMV (Acanthamoeba polyphaga mimivirus) megavirus isolated in 1992 from *Acanthamoeba (A.) polyphaga*, an amoeba living in the cooling tower water of a hospital in Bradford, England. The isolation took place during research into unusual cases of pneumonia in people in whom no infectious agents typical of pneumonia had been found [16]. Due to missing 16S rDNA analyses, that pathogen was at first mistaken for a *Gram*-*positive* bacteria and named *Bradfordcoccus* sp. [16]. It was only in 2003, after genetic tests were completed, that the pathogen was classified as a virus and named Mimivirus (short for “mimicking microbe”) on account of its resemblance to bacteria, or APMV, and due to its size, it was classified into NCLDVs, with a new family created for it—*Mimiviridae*, within which the genus Mimivirus was created [16]. The capsid of APMV has no outer viral envelope, has icosahedral symmetry and is circa 700 nm in diameter. On one of the capsid vertices, there is a pentagonal, star-shaped structure termed “stargate”, important in the initial phase of replication of this virus [1, 12, 18, 34–39]. It has at least two lipid membranes of different density and an internal membranous structure called a vesicle, which surrounds its genome and contains the enzymes necessary for infection of amoebae [18, 35–38]. Furthermore, the capsid surface was found to have fibril processes covered with 150-nm-thick fibres composed of peptidoglycan—an element typical of bacteria [49, 62, 82, 87, 88]. Such fibres are absent in other NCLDVs occurring in protozoa, although their presence in this giant virus is probably linked with the heterotrophic nature of its host—an amoeba that can mimic the bacteria on which protozoa feed [40]. This element of the giant virus structure is probably responsible not only for the positive result in the Gram staining but also for the protection of the virus against chemicals and for its adherence to amoebal cells during an infection [18, 35, 36]. It is suggested that this element supports and also conditions the adhesion of the Sputnik virophage to the surface of this giant virus during the infection of amoebae just at the time when they are “entering” the amoebae [36]. It is now assumed that the Sputnik virophage discovered in 2008 in the giant virus called ACMV (*Acanthamoeba castellanii mamavirus*) and also referred to as Mamavirus also infects the characterised giant virus APMV because, as has been recently accepted, the megavirus ACMV is a strain of the giant virus APMV [8]. Furthermore, it has been demonstrated that the ACMV Mamavirus, a “host” to the Sputnik virophage, is also infected by another virophage referred to as Sputnik 3 (Table 1).

Once APMV has penetrated amoebal cells by phagocytosis, it replicates; however, for the first 4–5 h of the replication (the eclipse phase), the phagocytised viral particles are only present in the amoebal phagosomes—but only until the stargate structure opens to enable translation of the viral DNA [18, 35, 41]. This leads to membrane fusion and virus release in the amoebal cytoplasm. This process resembles DNA segregation in bacteria [18, 35, 41]. It is assumed that the entire APMV replication cycle takes place in the amoebal cytoplasm, in the so-called “viral particle factory”, but it is partly dependent on the protozoon’s nucleus as there is evidence that nuclear agents take part in this process [15, 18, 19]. Whereas the genetic material of the newly formed APMV virions is packaged through the partly formed pores on the edge of the “viral particle factory” [42]. Research results have also proven that once an amoeba is infected by APMV, vesicles appear in its cytoplasm as soon as 2 h later. The vesicles probably transport the nuclear factors to the “viral particle factories” [19]. The final stage of APMV’s maturation, including the “assembly” of the peripheral fibril layer in its capsid takes place in the amoebal cytoplasm and its replication cycle ends with complete lysis of amoebal cells from which about 1000 descendant megaviruses APMV are released [42].

APMV’s genome is linear dsDNA, abundant in AT bases and as long as 1181,000 base pairs. It displays considerable diversity: as many as 1018 transcriptomes have been reported, including 979 genes, many of which have not been described nor have functions that are not found in viruses, like the encoding of translation-related proteins [14]. Moreover, six of the genes encode tRNA and 33 genes encode mRNA with an unknown function. Plus, APMV’s genome is capable of storing gene promoters [14]. It is also speculated that among APMV’s genes there are eight genes encoding the proteins involved in the translation and encoding an aminoacyl tRNA synthetase for arginine, cysteine, methionine and tyrosine, nine genes typical of NCLDVs encoding DNA polymerase and capsid proteins, as well as three genes encoding helicases, ATPase, thiol oxidoreductase, protein kinase and a transcription factor—all these factors are involved in the packaging of virions [3, 14, 15, 18, 19, 29, 30, 38, 43–50]. Furthermore, the APMV genome was observed to contain structures typical of other organisms, including structures responsible for tRNA translation and modification, DNA repair, protein folding and modification, nucleotide synthesis, amino acid and lipid metabolism and synthesis and polysaccharide and peptidoglycan metabolism [3, 4, 14, 29, 44, 46, 47, 50, 51]. It is assumed that these elements of the APMV genome condition its high mosaicity and this determines its instability. This feature may condition the significant extent of the range of its hosts—i.e. its infectious spectrum [2–4, 46, 47, 49].

The second giant virus of the Mimivirus genus, family *Mimiviridae* (Table 1), infecting protozoa, is the megavirus ACMV, referred to as Mamavirus. It was isolated in 2008 from the amoeba *A. castellanii* from the cooling tower water of a Paris hospital and is now recognised as a strain of the giant virus APMV [8]. An investigation of the megavirus ACMV found that in the end of its eclipse phase, a small “growth” appeared and apparently was an infectious agent for the virus. And on account of its similarity to bacteriophages that infect bacteria, it was termed a virophage—a “devourer” of viruses [8]. This virophage was named Sputnik (‘travelling companion’ in Russian), in honour of the first artificial satellite of the Earth [29]. A bit later, in 2013, it was shown that this giant virus ACMV is also infected by another virophage—the one called Sputnik 3 which was found in the amoeba *A. polyphaga* that does not live in water but in the soil in the territory of France (Table 1). Morphologically, the giant virus ACMV resembles the megavirus APMV as its capsid also has icosahedral symmetry, although the length of its genome is 1,191,693 base pairs. This means that this giant virus is larger than APMV by about 10,000 base pairs but both the viruses have as much as circa 90% of identical nucleotides [8, 44]. Moreover, it has been shown that ACMV’s genome contains an approximately 13,000 base-pair long segment at the 5′ end that is not found in APMV although the latter has a 900 base-pair long segment at the 3′ end which is typical of NCLDVs [44]. The discovery of the megavirus ACMV and the identification of the Sputnik virophage within the virus in 2008 as well as the identification of the Sputnik 3 virophage (Table 1) in 2013 were what caused the peculiar revolution in virology which resulted in the search for other megaviruses and their “parasites”—virophages, biological elements that had yet not been reported to be present in viruses, including giant viruses [52].

The third megavirus of the genus Mimivirus, family *Mimiviridae*, is the Lentille virus (Table 1), isolated in 2012 from the amoeba *A. polyphaga* that was identified in a contact lens solution used by a patient. In this megavirus, a new virophage was found and named Sputnik 2 [7, 17]. For this megavirus that also has dsDNA (the arrangement has not been determined), there is no information on the symmetry of its capsid but, interestingly, extrachromosomal DNA, abundant in GC pairs, was found in it. To date, such DNA has not been described as present in viruses. The GC pairs were defined as transpovirons (analogues of transposons in bacteria), the free forms of which replicate, just as the Lentille virus does, in the “viral particle factory” and, in addition, they accumulate in the Sputnik 2 virophage, too [7, 44]. Both the Sputnik 2 virophage in the Lentille virus and its transpoviron have been proven to integrate into almost anywhere in the chromosome of the giant virus Lentille, whereas the transpoviron associated with the virophage can undergo recombination with many other organisms [44]. The Lentille virus has also been reported to have a fragment of 7420 base pairs with a 24% content of GC pairs with identified 6–8 protein-coding genes, including two homologues for a virophage (Sputnik 2) [7].

The fourth megavirus of the genus Mimivirus, family *Mimiviridae*, is the giant virus Samba, isolated in 2014 from the amoeba *A. castellanii* from the water of the Negro River in Amazonia, Brazil. As is the case with other megaviruses in protozoa, this megavirus penetrates the amoeba by phagocytosis and is a “host” to the fourth described virophage, named Rio Negro (Table 1). The megavirus Samba is about 27–30 nm bigger than APMV, even though the latter is 440 nm in diameter [53]. The capsid of the megavirus Samba is structurally diverse and differs from the icosahedral symmetry. Like the giant virus APMV, Samba features a number of layers and its external fibrous layer is 30 nm deeper than that of the giant virus APMV. This means that Samba is the largest known virus of the genus *Mimivirus* [3, 53]. Like the giant virus APMV, Samba has been found to have a “stargate” structure [6]. The genome of the giant virus Samba is in the form of dsDNA (the arrangement has not been determined), measuring 1,213,607 base pairs, and is thus estimated to be circa 50,000 base pairs longer than the genome of the megavirus APMV [6]. Many of the ORFs specific to the giant virus Samba have been found in other viruses of the family *Mimiviridae*, including those which encode proteins involved in their translation or in DNA repair [6]. Furthermore, it has been demonstrated that 19 of its ORFs are involved in DNA replication, 10 are involved in DNA recombination, 14—in DNA repair and 5 of its ORFs—in aminoacylation of tRNAs, a process important for protein translation [6]. Also, 264 domains most likely involved in binding proteins and 200 domains associated with its catalytic activity have been identified in this megavirus [6]. It is also accepted that once the giant virus Samba has infected the amoeba *A. castellanii*, it takes over its “cellular apparatus” and creates a “viral particle factory” within its cytoplasm [6].

The other megaviruses from the genus Mimivirus of this family, making up the A line/group (Table 1) along with the characterised megavirus APMV, ACMV, Lentille and Samba, include four megaviruses isolated in 2015 from amoebae of the genus *Acanthamoeba* spp. and one giant virus found in 2016 in the amoebae *A. castellanii*—protozoa living in aquatic environments. The first of the megaviruses is Kroon recovered from amoebae living in a town lake in Brazil, the second one is Amazonia isolated from amoebae living in the Amazon River, the third is Oyster found in an amoeba from the water of an oyster farm located in Florianopolis on Brazil’s Atlantic coast and the fourth of the megaviruses is Niemeyer found in amoebae of a Brazil town centre lake. The fifth megavirus in this group is Mimivirus bombay, isolated in 2016 from the amoeba *A. castellanii* found in the waste water of Mumbai, India (Table 1). The first three viruses, i.e. Kroon, Amazonia and Oyster probably have a capsid with icosahedral symmetry [54] and are very much alike as the Kroon virus is 90–91% similar to the Amazonia and Oyster viruses, whereas the two latter are basically analogous to each other [54]. Despite this similarity, it is observed that, unlike the other two giant viruses, Amazonia and Oyster, the genome of the Kroon virus has no sequences coding for tryptophan but, like those two, it has a region coding for leucine, histidine and cysteine [54]. The genomes of these three megaviruses are of similar size: the Kroon virus has 1,222,932 base pairs, the Amazonia virus has 1,179,579 base pairs and the Oyster virus has 1,200,220 base pairs. Kroon is the largest of these viruses and among the nine viruses of the genus Mimivirus, family *Mimiviridae*, that have been described to date (Table 1). In contrast to these two giant viruses found in aquatic environments of Brazil and discussed above (Amazonia, Oyster), the Niemeyer virus, isolated, like the other viruses, from amoebae of the genus *Acanthamoeba* spp., lives, just as Kroon does, in amoebae living in a town centre lake in Brazil. Niemeyer’s capsid of 493 nm has icosahedral symmetry and its genome is in the form of dsDNA (the arrangement has not been determined), measuring 1,299,140 base pairs, coding for over 1003 proteins sized 100 to 2156 amino acids and containing three out of the four genes encoding aminoacyl tRNA synthetases for cysteine, methionine and tyrosine [55]. The last giant virus of the genus Mimivirus, family *Mimiviridae,* a member of the A line/group, is Mimivirus bombay (Table 1) which has a capsid with icosahedral symmetry and a size of 435 nm. Its genome’s length is 1,182,200 base pairs [56]. The characterised 5 megaviruses, i.e. Kroon, Amazonia, Oyster, Niemeyer and Mimivirus bombay, are no “hosts” to virophages (Table 1).

Whereas the B line/group is made up of other three megaviruses of the genus Mimivirus, family *Mimiviridae*, infecting protozoa, which are Moumouvirus—Acanthamoeba polyphaga moumouvirus, isolated in 2008 from the amoebae *A. polyphaga* from the water of a cooling tower in France; Moumouvirus monve, also isolated in 2008 but from amoebae of the genus *Acanthamoeba* spp. living in the water of a cooling tower of a hospital in France and the giant virus Saudi moumouvirus, isolated from the waste water of a hospital located in Jeddah, Saudi Arabia. As is the case with Kroon, Amazonia, Oyster, Niemeyer and Mimivirus bombay, no virophages are found in these three viruses in question (Table 1). It has been shown that the symmetry of the capsid of Moumouvirus—Acanthamoeba polyphaga moumouvirus is icosahedral, 420 nm in size, and covered with a 100-nm-thick fibrous layer, and that its dsDNA genome (the arrangement has not been determined) is composed of 1,021,348 base pairs [14, 30]. Like in the case of the giant virus APMV, some of the Moumouviruses—Acanthamoeba polyphaga moumouvirus have been found to have a “stargate” vertex [30]. This virus codes for 930 proteins, among which only 879 have homologues of viruses of the family *Mimiviridae*. As many as 702 of these homologues, with amino acid conformity at 62%, have been discovered in Megavirus chilensis (of the same genus and the same family) (Table 1). Moreover, this giant virus has been proven to also have 27 genes homologous to bacteria [30]. Its replication cycle in the “viral particle factory” is similar to that of APMV and Megavirus chilensis [30]. The other megavirus in the B line/group—Moumouvirus monve (Table 1) is closely related to the characterised Moumouvirus—Acanthamoeba polyphaga moumouvirus as they share certain features. The third megavirus in the B line/group—Saudi moumouvirus (Table 1) has a capsid with icosahedral symmetry and a size of 500 nm. Its genome is 1,046,087 base pairs long and codes for 868 ORFs, the sizes of which range between 54 and 2914 amino acids [57]. Apart from 42 proteins, presumably sized below 100 amino acids, its genome codes for 40 genes that lack similarity to other sequences making up the protozoal megaviruses presented in the NCBI database [1].

Further five giant viruses of the genus Mimivirus, family *Mimiviridae*, infecting protozoa, are Courdo11, Megavirus chilensis, Mont1, Shan and the megavirus LBA111—all making up the C line/group (Table 1). The megaviruses of this line/group, except for Mont1 that lives in the amoeba *A. polyphaga*, live in amoebae of the genus *Acanthamoeba* spp. (Table 1). The giant virus Courdo11 has been proven to be present in an amoeba living in a river of the French area of Le Peyron (Table 1). Its capsid is probably icosahedral in symmetry and about 450 nm in size, and its dsDNA (the arrangement has not been determined) is 1,245,654 base pairs long [58]. This megavirus also shows considerable similarity to the giant virus Megavirus chilensis as many as 97.5% of its proteins are homologous to *M. chilensis*. It also shows 95.5% protein homology to the megavirus LBA111, and as many as 73.7% of its genes are orthologous to *M. chilensis*, while 73.45% of them—to the megavirus LBA111 [58]. Also, nearly 34% of its genes are orthologous to the giant virus APMV of the A line/group and 39% of them—to Moumouvirus virus of the B line/group [58]. The other giant virus, Megavirus chilensis of the C line/group (Table 1), has been isolated from an amoeba analogous to that in the case of the Courdo11 virus but living in seawater of Chile (Table 1). Megavirus chilensis is the largest virus of the genus Mimivirus, family *Mimiviridae*, infecting protozoa as its genome’s size is 1,259,197 base pairs [43, 44, 59]. It has a capsid with icosahedral symmetry and is 23% similar to APMV and 50% similar to other megaviruses of the genus *Mimivirus*, family *Mimiviridae* [43, 44, 59], although its key genes are assumed to be very specific to giant viruses of the family *Mimiviridae* [43, 45]. Apart from encoding four homologues of aminoacyl tRNA synthetase, analogous with those in the giant virus APMV, this virus also encodes three other homologues [43, 45]. The third giant virus, Mont1 of the C line/group, of the genus *Mimivirus*, family *Mimiviridae*, has been found in the amoeba *A. polyphaga* in samples of Tunisian soil (Table 1). Its capsid symmetry is analogous to that of the giant virus Megavirus chilensis and its size is determined to be around 500 nm. Its molecule is surrounded by fibres [60]. The difference between the giant virus Mont1 and the other two megaviruses of the C line/group, i.e. Courdo11 and Megavirus chilensis, is that Mont1 has the fifth described virophage, named Zamilon (Table 1). Unlike Sputnik, Sputnik 2, Sputnik 3 and Rio Negro, this virophage has a barely discernible effect on the replication of the megavirus Mont1—it rather only makes its capsid shape irregular [60, 61]. However, Mont1, which is a “host” to Zamilon, has been found to have a defence system against virophages, called MIMIVIRE. It is very similar to the CRISPR-Cas system and is quite common among bacteria and archaea. It is also found in megaviruses infecting protozoa of the A and B line/groups of the genus Mimivirus, which would suggest that this system may be specific to other giant viruses of this family [9]. Two other megaviruses, Shan and LBA111, which also belong to the C line/group of the family *Mimiviridae*, genus Mimivirus (Table 1), have been isolated, as previously mentioned, from amoebae of the genus *Acanthamoeba* spp., but they are hosts to humans (Table 1). The giant virus Shan has been isolated from an amoeba living in the faeces of a 17-year-old girl with pneumonia, whereas the LBA111 virus has been isolated from an amoeba of the genus *Acanthamoeba* spp., but found in a bronchoscopy specimen from a 72-year-old woman with pneumonia (Table 1). The Shan virus has a capsid with icosahedral symmetry, is 640 nm in size, has a very thick layer of fibres and a “stargate” structure [62], whereas the LBA111 virus also has a capsid with icosahedral structure but, with a size of 554 nm, it is a bit smaller. However, its genome (the arrangement has not been determined) has 1,230,522 base pairs [14, 26].

Among the megaviruses of the family *Mimiviridae*, infecting protozoa, there is, apart from the genus Mimivirus, another genus that has been described and named Cafeteriavirus. It is represented by one species only—*Cafeteria roenbergensis* virus (CroV) that does not infect amoebae, but rather flagellates (*Cafeteria roenbergensis*) living in the Gulf of Mexico. In this megavirus, the sixth virophage, Mavirus, has been found (Table 1). CroV is an evolutionary intermediate between DNA viruses and eukaryotic Maverick/Polinton DNA transposons, which among eukaryotic viruses, are prevalent elements that encode virus-like genes and for this reason they have been termed “polintoviruses” as they represent the family of endogenous viral elements among eukaryotes [44, 63]. The giant virus CroV has a capsid with icosahedral symmetry with a diameter of 300 nm and an outer lipid membrane but no line/group has been determined for this megavirus [Table 1]. Its genome is composed of 730,000 base pairs; however, its central part is made up of 618,000 base pairs, among which 544 pairs code for proteins [5, 63, 64]. At both ends of its genome there are large, recurring regions which possibly are its protective parts that code for proteins related to telomeres in eukaryotes [64]. The genome of this giant virus is rich in AT pairs (ca. 70%), which is reflected in the distribution of codons and in the general composition of amino acids [64, 65]. Its codons rich in AT pairs are more preferred than those rich in CG pairs [64]. The most common amino acids in the genome of this virus are lysine, isoleucine, asparagine and leucine, each of which makes up about 10% [64]. This virus has only 5 of the 47 main genes that are universal among NCLDVs [5]. Among its 14 open reading frames (ORFs), there are no genes of the 47 genes universal among NCLDVs, mentioned above, and no genes for the four proteins involved in DNA replication, recombination and repair or in nucleotide metabolism, but RNA ligase and dUTPase, which are absent in, e.g. the giant virus APMV, have been proven to be present in this virus [5]. It is also accepted that approximately half of the genes of the giant virus CroV are similar to the proteins present in eukaryotes, bacteria, archaea and other giant viruses [64]. Whereas among the 177 NCVOGs (Nucleo-Cytoplasmic Virus Orthologous Groups) present in viruses of the NCLDV group that includes protozoal megaviruses, 59 do not exhibit the same presence/absence pattern in the giant virus CroV and in APMV—this pattern only appears in four cases, i.e. in those of dUTPase, adenine-specific DNA methyltransferase, ubiquitin and RNA ligase as these NCVOGs are present in the megavirus CroV but absent in the megavirus APMV, while the other 55 NCVOGs are present in APMV but absent in CroV [5]. Although the majority of the coding sequences in the megavirus CroV have an unknown function, a hypothetical function can be specified for 32% of them; however, some of them are not coded by any other giant viruses in protozoa [64]. The replication of this giant virus is mainly based on the mechanism of translation of its hosts’ proteins, hence it is not typical to find genes specific to the megavirus CroV [64]. The giant virus CroV encodes isoleucyl-tRNA synthetase and presumably also homologues of eukaryotic factors involved in translation initiation, such as eIF-1, eIF-2α, eIF-2β/eIF-5, eIF-2γ, eIF-4AIII, eIF-4E and eIF-5B [64]. Additional 22 tRNA genes and two potential tRNA-modifying enzymes, i.e. tRNA pseudouridine 5S synthetase and tRNA lysine synthetase that contribute to the rapid rise in the number of components during translation of proteins have been identified in this giant virus [30]. However, some tRNA genes of the megavirus CroV have been reported to be also present in bacteriophages and eukaryotic viruses, e.g. in *Phycodnavirus* sp. infecting algae [64]. Research has also showed that four tRNA synthetases of this virus, along with several potential factors involved in translation initiation are also present in other giant viruses of the genus *Mimivirus*, family *Mimiviridae* [64]. These facts suggest that the giant virus CroV codes for similar viral complexes for modification and regulation of the host’s translation system, which probably leads to a “lifestyle” that is less dependent on the constituents of the host’s cells than is the case with other giant viruses infecting protozoa [64]. Proteomic analyses of CroV have also [66] shown that it is a complex repertoire of proteins as it contains not only structural proteins but also a broad set of proteins involved in cellular processes, including those involved in mRNA synthesis, DNA repair, disulphide bridge formation as well as in producing different proteases and phosphatases. An ion channel protein having mechanical action has also been found in this virus [66]. Phylogenetic reconstructions based on ribonucleotide reductase (RNR), family B polymerase (DNApol), proliferating cell nuclear antigen (PCNA), FLAP endonuclease (FEN) and on the transcription factor II B (TFIIB) indicate a similarity between the proteins of CroV and those of the giant virus APMV and other viruses of the *Mimiviridae* in respect of TFIIB and the transcription factor related to the RNA polymerase II reinitiation complex [5]. Whereas phylogenetic assessment of DNA-dependent RNA polymerase II (RNAP II) provides evidence to support the theory that RNAP II of this virus is more closely related to the clade of viruses of the family *Irido-/Ascoviridae* [5]. The CroV virus replicate in large cytoplasmic “viral particle factories” produced in its host *C. roenbergensis* where not only the replication of its DNA but also the transcription and the accumulation of its new particles take place [65].

Other megaviruses of the family *Mimiviridae*, infecting protozoa, are two nameless giant viruses, about which relatively little is known. They were discovered, one after the other, in 2013 and 2015 (Table 1). The first of these giant viruses (Table 1) was found in the water of a lake in Antarctica, while the second—in the contents of a sheep’s rumen. These two megaviruses have been proven to have the ALM virophage and the RVP virophage (Table 1) but, as was the case with the CroV megavirus, no line/group has been determined for them (Table 1).

Four other megavirus species make up a group of giant viruses within the family *Mimiviridae*, about which little is known, too. The species belong to the genus Klosneuvirus that is represented by the species/isolate Klosneuvirus, and to the genera Catovirus, Hokovirus and Indivirus (Table 1). From among these viruses, described in 2017, the megavirus Klosneuvirus was discovered in amoebae of the genus *Acanthamoeba* spp., living in the waste water of a sewage treatment plant in Klosterneuburg, Austria. This giant virus has a dsDNA genome (the arrangement has not been determined) sized 1.57 Mb and contains 28.6% of GC pairs [67, 68]. Its translational apparatus is very complex, and along with giant viruses of the genus Mimivirus and the giant virus CroV, it makes up the basic genetic clades within the family *Mimiviridae* [67]. Whereas the other three species of the genus Klosneuvirus have been identified as isolates solely on the basis of genetic tests and are represented by Catovirus, the genome of which has a size of 1.53 Mb and 26.4% of GC pairs, by Hokovirus with a genome of 1.33 Mb and 21.4% of GC pairs, and by Indivirus that has a genome with a size of 0.86 Mb and 26.6% of GC pairs [68]. For the viruses of this genus, just as for CroV and the two nameless viruses, no names or line/groups have been determined.

The latest megavirus genus within the family *Mimiviridae*, described in 2018, is Tupanvirus, represented by two species/isolates infecting the amoebae *A. castellanii* and *Vermamoeba vermiformis* (Table 1). One of them has been identified in protozoa living in a salt lake in the Nhecolândia region of Brazil, while the other—in the Atlantic Ocean near Campos dos Goytacazes, Brazil, at a depth of 3000 m (Table 1). Like all the three other megavirus genera that have been identified within the family *Mimiviridae* to date, i.e. Mimivirus, Cafeteriavirus and Klosneuvirus, and like one nameless genus within the family *Mimiviridae* (Table 1), tupanviruses have its genetic material in the dsDNA form (the arrangement has not been determined) and the size of their capsids is around 450 nm. Interestingly, their virions feature a long cylindrical tail which measures about 550 nm and is actually the longest tail in the virosphere [67]. For the Tupanvirus of the salt lake it has been shown that it is 1,439,508 base pair long and has 1276 reading frames, whereas the Tupanvirus living in the ocean is 1,516,267 base pair long and has 1425 reading frames. However, the GC pair content within the genome of both these megaviruses is estimated to be about 28% [67]. Furthermore, it has been demonstrated that as many as 5165 genes of giant viruses of the genus Tupanvirus are only specific to this genus and 775 of the genes are not found in other viruses of the family *Mimiviridae* [67]. Finally, megaviruses of this genus have been shown to have the most complete transcriptional apparatus in the whole virosphere [67]. As was the case with Cafeteriavirus, two megaviruses without a generic or specific name and with the genus Klosneuvirus, no line/group has been determined for tupanviruses but they have been shown to be 10% similar to viruses of the A line/group, 18% similar to B and 14% similar to C [67].

Although the presented four genera of megaviruses and one nameless genus from the family *Mimiviridae,* infecting protozoa (amoebae, flagellates) (Table 1), have a number of specific features, they also share a number of elements with the first described giant virus, that is APMV [16]. These characterised giant viruses of the family *Mimiviridae* are common elements of nature as they comprise a large percentage of the genomes present in protozoa living in aquatic environments and in the soil, as well as in algae, sponges and corals living in waters [15, 22, 25]. Surprisingly, these megaviruses are also found in amoebae living in invertebrates (molluscs: mussels, insects) and in vertebrates (humans, sheep) (Table 1). They constitute—and this is very important—a component of the human and animal microbiota as they have been found in the faeces of healthy people and in the gastrointestinal tract of healthy animals, as well as in bronchoscopy specimens, including those from patients with pneumonia or other pulmonary diseases [23, 24, 26, 31, 66]. Moreover, it is a very interesting biological fact about giant viruses infecting protozoa that when the best described virus of the family *Mimiviridae*, APMV, a “host” to the Sputnik virophage infects a human, brings on specific activation in its immune system, mainly in macrophages [24]. Through phagocytosis, this virus increases their production of itaconic acid which primarily increases synthesis of IFN-β that has an antimicrobial effect against, inter alia, APMV [24]. Furthermore, this activation also leads to stimulation of IRG1 genes (immune responsible genes 1) which results in stimulation of many other components of the immune system, including the interferon system in respect of IFN-β and of the said itaconic acid. According to researchers [24], this is a new (previously not reported) way in which viruses exert an effect on the immune system in mammals, including humans [24].

### Giant viruses of the family *Marseilleviridae*

Megaviruses of this family are represented by 11 species constituting one genus—*Marseillevirus* (Tables 2 and 86). These giant viruses infect not only amoebae living in aquatic environments but also those living in insects and mussels, as well as in amoebae in the faeces of healthy humans and in amoebae found in tissue samples from humans (Table 2). Nine of these 11 megavirus species have been classified into five lines/groups (A, B, C, D, E). No line/group has been created for two species (Table 2). No virophages have been found in any of the 11 megaviruses (Table 2).

The first megavirus of this family is *Acanthamoeba polyphaga marseillevirus* (APMaV), described in 2007 as living in the amoeba *Acanthamoeba polyphaga,* found in the cooling tower water of a hospital in Paris, France (Table 2). The discovery of this virus, along with its specific features that distinguish it from the known megaviruses of the family *Mimiviridae*, living in protozoa, started a new family *Marseilleviridae* [24]. There is evidence that the capsid of this virus (APMaV) has icosahedral symmetry with a diameter of 250 nm and 10-nm-thick outer envelope in which there are fibril processes with about 12-nm-long round ends. As is the case with viruses of the family *Mimiviridae*, this giant virus infects amoebae by phagocytosis. However, its total replication cycle is much shorter compared to that in giant viruses of the family *Mimiviridae*, as it lasts 5 h only [69]. The APMaV’s entry to an amoeba lasts 30–60 min and just after that time, a “viral particle factory” is being created in the protozoan’s cytoplasm, near the nucleus, the genetic material is being “assembled” and new APMaV virions are being produced [69]. After the megavirus’ entry to the amoeba, the most intense changes occur between 30 min and 2.5 h in their nucleus [69]. Unlike in megaviruses of the family *Mimiviridae*, the genetic material of APMaV is circular dsDNA with 44.7% content of GC pairs sized 36,000 base pairs, containing 475 described ORFs, thus suggesting its dense packing [34]. Its genome displays immense diversity as only 11% of its genes are typical of NCLDVs, 11% of the genes are of bacterial or bacteriophagic origin and as many as 19% are of eukaryotic origin [34]. It is astonishing to note that in this megavirus there are genes encoding histone-like proteins and histone doublets which have only been found in eukaryotic organisms so far [34]. A variant of this megavirus was discovered in 2013 when human blood was investigated. It was then described and named “giant blood *Marseillevirus*”. It has 617 DNA coding sequences, 436 of which are homologous to the giant virus APMaV and 33 are similar to the megavirus Lausannevirus which also is a member of the family *Marseilleviridae* [70].

Other giant viruses of the family *Marseilleviridae,* infecting protozoa, include Cannes 8, described in 2013 and found in an amoeba of the genus *Acanthamoeba* spp., from the water of a cooling tower in Cannes, France (Table 2), and Senegalvirus (Table 2), also isolated from amoebae of the genus *Acanthamoeba* spp., found in the faeces of a healthy man living in Senegal—the place of this discovery was very surprising [66]. This family also includes Melbournevirus which is also found in amoebae of the genus *Acanthamoeba* spp. but in those living in a freshwater pond in Melbourne (Table 2). These three giant viruses (Cannes 8, Senegalvirus, Melbournevirus) and the aforementioned Acanthamoeba polyphaga *marseillevirus* (APMaV) of the family *Marseilleviridae* belong to the A line/group (Table 2). Research into the megavirus Cannes 8 has shown that it has a capsid with icosahedral symmetry and is similar to viruses of the genus *Marseillevirus* (Table 2). Like in APMaV, its genome is circular dsDNA with a length of 374,041 base pairs which encode 484 proteins containing from 50 to 1537 amino acids, among which 380 (79%) are orthologues of the megavirus APMaV, while 272 (56%)—orthologues of Lausannevirus, whereas the remaining amino acids are common to both the megaviruses, i.e. APMaV and Lausannevirus [71]. The genome of Cannes 8 has been proven to demonstrate high collinearity as it shares as many as 96% of orthologues with the genome of APMaV and as much as 2/3 of its gene repertoire is specific to megaviruses of the family *Marseilleviridae* [71]. However, another megavirus of the family *Marseilleviridae* (Table 2), Senegalvirus, also has, just as APMaV and Cannes 8 of this family do, icosahedral symmetry with a diameter of 210 nm, and its genome is 373,000 base pair long [14]. Whereas the megavirus Melbournevirus of this family (Table 2), which has also been found in amoebae of the genus *Acanthamoeba* spp., but in those living in a freshwater pond in Melbourne, Australia, has icosahedral capsid symmetry, too, a size of 200 nm and no fibres [72]. The genome of this virus is in the form of dsDNA (the arrangement has not been determined), with a length of 369,360 base pairs and with GC content of 44.7%, encoding 403 ORFs [6, 41]. Its coding sequences occupy 86.7% of its genome, and among them there are short non-coding sequences containing about 122 nucleotides [72]. This giant virus (Melbournevirus) is 98% identical with the megavirus APMaV and the virus Cannes 8, which means strong similarity between them as viruses found in habitats that are remote from one another [72].

The next megavirus member of the family *Marseilleviridae*, infecting protozoa but making up the B line/group is *Lausannevirus* isolated in 2011 from the amoeba *Acanthamoeba castellanii* (Table 2) found in the Seine (France) [14, 73]. This virus is characterised by a narrow host range. Its capsid has 200 nm icosahedral symmetry and its genome is 346,000 base pairs long and contains 450 genes. Genetically, it is 89% similar to the megavirus APMaV [73, 74]. Its genome is in the form of circular or linear dsDNA which is a very surprising fact, and in addition, it encodes three proteins with histone folds, including two doublets which are similar to eukaryotic and archaeal histones [73].

Further viruses of this family, infecting protozoa, are two giant viruses making up the C line/group (Table 2). One of them is the megavirus Insectomime, isolated in 2013 from amoeba of the genus Acanthamoeba spp., living on the larva of the insect *Eristalis tenax* (Table 2), while the other is the megavirus Tunisia virus found in 2014 in an amoeba of the *Acanthamoeba* spp., too, but living in the water of a fountain in Tunisia [75]. The genome of Insectomime is in the form of dsDNA (the arrangement has not been determined) measuring 386,000 base pair that encode 477 proteins containing from 46 to 1643 amino acids, 435 of which are present in APMaV and Lausannevirus [25]. The capsid of this virus has icosahedral symmetry sized 225 nm [14, 76] and is most closely related to the megavirus Tunisia virus, with which it shares 446 orthologues [76]. Like in Insectomime, the capsid symmetry in Tunisia virus is icosahedral, with a diameter of 250 nm, and its genome is in the form of dsDNA (the arrangement has not been determined), containing 380,011 base pairs that encode 484 proteins and make up 21 ORFs [76].

Another giant virus of the family *Marseilleviridae* is Brazilian *marseillevirus*, described in 2016, infecting amoebae of the family *Acanthamoeba* spp. that live in the waste water of a sewage treatment plant located on Lake Pampulha in Brazil. Brazilian *marseillevirus* makes up the D line/group of this family (Table 2). Its genome is in the form of dsDNA (the arrangement has not been specified), containing 362,276 base pairs, with the GC pair content being 43.3%. The genome encodes 491 ORFs and 3 histones (H2A, histone fusion protein H2B/H2A and histone H3), similar to those occurring in the megavirus Lausannevirus [77]. There is no information on this virus’ capsid symmetry, although it is suggested to be icosahedral just as in other viruses of the family *Marseilleviridae* [77]. The next megavirus is Tokyovirus found in amoebae living in the mud of the Arakawa River in Tokyo, Japan. It makes up the E line/group of the family *Marseilleviridae* (Table 2). This megavirus has icosahedral capsid symmetry with a diameter of 200 nm [65]. Its genome is in the form of dsDNA (the arrangement has not been specified) measuring between 360,000 and 370,000 base pairs that have 487 ORF coding sequences [78]. It is most closely related to APMaV, Cannes 8 and Melbournevirus which make up the A line/group within this family (Table 2).

Further giant viruses of this family, infecting protozoa, are Golden *marseillevirus* and *Marseillevirus lymphoma*, described in 2016. They were found in two completely different environments. No line/group has been determined for them (Table 2). Golden *marseillevirus* was identified in the amoeba Acanthamoeba polyphaga living in the mussels *Limnoperna fortunei* that live in a lake in southern Brazil (Table 2), whereas the megavirus *Marseillevirus lymphoma* was identified in an amoeba of the Acanthamoeba spp., in lymph node specimens from a patient with Hodgkin’s lymphoma (Table 2). Golden *marseillevirus* has icosahedral capsid symmetry with a diameter of 200 nm and its genetic material is, as is the case with APMaV and Cannes 8, in the form of circular dsDNA containing 360,610 base pairs that encode 483 ORFs. In phylogenetic analysis, the genome differs from the other viruses of the family *Marseilleviridae* [79]. In contrast, the capsid and genome structures of *Marseillevirus lymphoma* are similar to those of other megavirus of this family [79].

The presented 11 virus species of the genus *Marseillevirus*, family *Marseilleviridae*, infecting amoebae only but living in aquatic environments as well as in eukaryotic organism (humans, mussels, insects) indicate new facts about phylogeny of megaviruses in protozoa as, inter alia, it has hitherto been assumed that megaviruses of this family make up 3 lines/groups only: A, B and C (Tab. 2), whereas for the isolated megavirus Brazilian *marseillevirus*, the D line has been proposed, for *Tokyovirus*—the E line and for Golden *marseillevirus* and *Marseillevirus lymphoma*—no line/group has been determined (Table 2). These facts about their phylogenetics and the identification of these viruses in amoebae living in untypical places like, apart from aquatic environments, also in the excrement (faeces) of healthy people and in neoplastic lesions in humans, as well as in invertebrates (mussels, insects) (Table 2), indicate that giant viruses actually can be the “thing” that makes it necessary to redefine the existing nature-related truths, at least those related to virology [71, 78].

### Giant viruses making up no families

These megaviruses infecting protozoa (amoebae) living in aquatic environments and making up no families, are represented by 17 species/isolates of these viruses grouped into four genera (Pandoravirus, Pithovirus, Faustovirus and Mollivirus) and one species named Kaumoebavirus, without a generic name. Among these giant viruses, only nine species/isolates have been classified into a line/group and, as is the case with 11 virus species of the family *Marseilleviridae*, they are no “hosts” to virophages (Table 3).

The first megaviruses of this group are three species of the genus Pandoravirus (which has its name after the mythological Pandora’s box due to its shape that resembles a box, i.e. *P. salinus*, *P. dulcis* and *P. inopinatum* for which no line/group has been determined (Table 3). The first two species (*P. salinus* and *P. dulcis*) were identified in 2013 in amoebae of the genus *Acanthamoeba* spp. (Table 3) and these are megaviruses which considerably differ in terms of morphology and genetics from the previously described giant viruses of the families *Mimiviridae* and *Marseilleviridae* (Tables 1 and 2), however, the giant virus *P. salinus* was found in amoebae living in the deposit of river in the central part of Chile’s coast, whereas *P. dulcis* was isolated from amoebae living in the mud of a freshwater pond located near Melbourne, Australia (Table 3). The third megavirus of the genus Pandoravirus is *P. inopinatum* (Table 3), isolated in 2015 as an endosymbiont of amoebae of the genus *Acanthamoeba*, strain LaHel that was found in a conjunctiva of a patient with keratitis [21]. These two first megaviruses (*P. salinus* and *P. dulcis)* are so large that they can be observed under a light microscope. They are characterised by a big oval capsid (500 × 1000 nm) with icosahedral symmetry and a three-layer outer envelope with pores on one side which allow them to bring their DNA in the host’s cytoplasm [42]. These viruses get into amoebae by phagocytosis and then, after release of its genetic material, they disorganise the amoebal nucleus where their genetic material replicates. This distinguishes them from viruses of the families *Mimiviridae* and *Marseilleviridae* which replicate in the cytoplasm of protozoa [80]. The time they need for the replication and release of new virions in the amoeba is 8–10 h of infection [80]. Their genetic material is linear dsDNA (the arrangement has not been determined), rich in GC bases [80]. However, the genome of *P. salinus* contains 2,450,000–2,470,000 base pairs and encodes 2556 potential sequences, including proteins, except for capsid proteins [14, 81]. Whereas the genome of *P. dulcis* has been determined to be 1,910,000 base pairs long and to have 1502 potential sequences that can encode proteins [20]. Research into the genomes of these megaviruses has shown that particularly genes of *P. salinus* largely remain typical of megaviruses infecting protozoa, with the greatest number of genes conditioning DNA replication, transcription and nucleotide synthesis [42]. Whereas the genome of the megavirus *P. inopinatum* contains 2,240,000 base pairs, encodes 1902 proteins and is 85% identical with *P. salinus* and 89% with *P. dulcis* [14, 81].

Other giant viruses that infect protozoa and do not make up any family are three species of the genus Pithovirus for which, just as for megaviruses of the genus Pandoravirus, no line/group has been determined (Table 3). The first one of them is *Pithovirus sibericum* identified in 2014 in an amoeba of the genus *Acanthamoeba* spp. living in 30,000-year-old Siberian permafrost in Chukotka, Russia [35, 82]. Like pandoraviruses, this megavirus is oval in shape but its capsid is larger than them as it is 1500 nm long and circa 500 wide, and, in addition, it is enclosed by a 60-nm-thick coat composed of 10-nm-long strips arranged in parallel [35]. At the vertex of this giant virus, there is a distinctive process built of strips spaced 15 nm apart and built of an internal membrane and an external one [35]. Despite the lack of information on capsid symmetry of this megavirus, it is assumed to most likely have icosahedral symmetry [16, 35, 71, 83]. Its genetic material is linear dsDNA, mostly formed by AT pairs [35]. Despite the large size of its capsid, the genome of this virus is relatively small as it only contains 610,000 base pairs which only encode 467 proteins. However, the genome is characterised by frequent repeats of palindrome sequences and of elements of the MIMIVIRE system which has also been found in the giant virus Mont1 of the family *Mimiviridae* [35]. *Pithovirus sibericum* replicates after entering an amoebal cell by phagocytosis in a “viral particle factory”; however, the nucleus of the infected amoeba does not change, as opposed to the replication of pandoraviruses during the infection of amoebae and the entire replication cycle of this and other giant viruses of this genus (Table 3), from infection to complete lysis, takes 15 h [42]. This virus is not similar to giant viruses of the families *Mimiviridae* (Table 1) and *Marseilleviridae* (Table 2), infecting protozoa, or to other giant viruses making up no families (Table 3), but it very similar to viruses of the genus *Iridovirus,* family *Iridoviridae* [35]. The second virus of the genus Pithovirus is *Pithovirus massiliensis*, found in 2015, also in an amoeba of the genus *Acanthamoeba* spp. that lived in the waste water of La Ciotat, France. As it is accepted, this virus probably is an analogue of the megavirus *P. sibericum* (Table 3) as they both share 93.5% of genome orthologues [84]. The genome of *Pithovirus massiliensis* has 683,000 base pairs, including GC pairs, encodes 520 genes and is 35% analogous to the giant virus *Pithovirus sibericum* in terms of genetics [84]. It is assumed that *Pithovirus massiliensis and Pithovirus sibericum* are good research forms, on the basis of which one can find out about the pace of evolution of viruses, including megaviruses [84]. The third giant virus of the genus *Pithovirus* is Cedratvirus (Table 3), isolated in 2015, also from amoebae of the genus *Acanthamoeba* spp. but those living in seawater at the Algerian coast [81]. Its shape resembles the citron fruit and for this reason, it has been named Cedratvirus [81]. The virus is oval but, as opposed to the two pithoviruses described above (*Pithovirus sibericum* and *Pithovirus massiliensis)* (Table 3), on both of its vertices there are distinctive processes—“corks” [81]. It is supposed that its capsid has icosahedral symmetry [81, 83] and its genome is 589,000 base pairs long. It shares 104 genes with *Pithovirus sibericum* and 193 genes with *Pithovirus massiliensis* [81]. Even though Cedratvirus is genetically very similar to *Pithovirus sibericum* and *Pithovirus massiliensis*, it is marked by another genome organisation and, in addition, it encodes proteins that have different functions [81]. It is also suggested [81] that the discovery and characterisation of *Pithovirus sibericum*, *Pithovirus massiliensis* and Cedratvirus (Table 3) has formed the basis for claiming that the genus Pithovirus can also be represented by other megavirus species.

The fourth genus of megaviruses infecting protozoa and creating no families is Faustovirus represented by 9 species/isolates (Table 3) which were described in 2015 and which make up 4 lines/groups (E9, M, D, L). It is assumed that the average length of their genomes is 467,592 base pairs [85]. All the 9 species/isolates of these viruses (Table 3) infect the amoebae *Vermamoeba vermiformis*, not *Acanthamoeba* spp. as is the case with viruses of the genus Pandoravirus and Pithovirus, and these protozoa are the most frequently isolated unicellular organisms living in the human environment [86]. Most is known about 4 of these species/isolates, i.e. about E9, E12, E23 and E24 which have been found in an amoeba living in waste water of Marseille [86] and forming the E9 and M lines/groups (Table 3). Other 4 megavirus species/isolates of this genus, i.e. D5a, D3, D5b and D6 have been identified in waste water near Dakar, Senegal. They make up the D line/group (Table 3). Another species/isolate of the genus Faustovirus is Faustovirus Liban, isolated from amoebae living in seawater of Lebanon (Table 3). All the 9 species/isolates of megaviruses of the genus Faustovirus (Table 3) get to the amoebae V*ermamoeba vermiformis* by phagocytosis, as is the case with other giant viruses infecting protozoa, and just after 2–4 h of infection, descendant virions can be found in the phagosome of these amoebae [86, 87]. The replication cycle of these giant viruses is similar to that of viruses of the families *Mimiviridae* and *Marseilleviridae* and takes place in “viral particle factories” which are located in the cytoplasm of the amoebae, near their mitochondria [12, 87]. With regard to these 9 Faustovirus species/isolates, most is known about their morphological and genetic features, as well as about replication of Faustovirus E12 (Table 3), the capsid of which has icosahedral symmetry with about 200 nm in diameter and its surface is free from fibres [86, 87]. The replication of Faustovirus E12 after the infection of the amoeba results in a number of changes, the most discernible of which is the amoebal nucleus losing its spherical shape [86, 87]. Research results have also proven that cell lysis in amoebae takes place between 18 and 20 h following their infection, which is a bit later compared to other giant viruses infecting protozoa [86, 87]. Like the genomes of the APMaV, Cannes and Golden *marseillevirus*, the genome of Faustovirus E12 is in the form of double-stranded circular DNA containing 460,000 base pairs and presumably encoding 451 proteins, of which only 164 are proteins conditioning viral functions [86, 87]. Furthermore, it has been demonstrated that this giant virus is most closely related to the African swine fever virus of the family *Asfarviridae* [28] but its genome is characterised by higher mosaicity and, apart from this, about two-thirds of its genes have no homologues in other giant viruses described to date [86, 87]. There is an unusual and peculiar fact about megaviruses of the genus Faustovirus. Namely they were also isolated from flies, which indicates that these insects are responsible for, inter alia, spreading a number of germs, including haemorrhagic fever viruses; they are also carriers of these giant viruses [28]. Researchers [28] claim that these faustoviruses can colonise rodents and cattle. However, these viruses have also been found in the serum of healthy people as well as in people with unexplained fever. It is therefore accepted that research into these viruses, if only for collection of data on their biology and infectious capacity, is becoming a necessity.

Among the giant viruses infecting protozoa and making up no families there is the genus Mollivirus with 1 species—*Mollivirus sibericum* (Table 3) which was identified in 2014 in the amoebae *Acanthamoeba castellanii*—in the same sample of permafrost from the Kamchatka Peninsula region in the Russian Far East in which the giant virus *Pithovirus sibericum* was identified [35]. This virus presumably has an icosahedral capsid shape with a diameter of 500-600 nm and a coat consisting of two layers of different thickness [71, 83, 88]. The external layer seems to be made of 30–40 nm structures cutting the coat’s surface, whereas the internal lipid layer is 12–14 nm thick and is made of a fibre mesh similar to that described in the case of *Pithovirus sibericum*, although *Mollivirus sibericum* is genetically very diverse compared to *Pithovirus sibericum* [88]. Its genome is in the form of linear dsDNA containing about 6,51,000 base pairs that encode 523 genes responsible for the formation of proteins and 3 tRNA coding genes [14]. Among its genes, 18% are homologous to viral genes and as many as 14% are homologous to eukaryotic genes, while 3% are similar to bacterial genes [42]. It has been found that one of the main genes encoding proteins of *Mollivirus sibericum* is very closely related to proteins characterised in the cases of giant viruses as well as to proteins of the amoeba *A. castellanii* that are probably descended from other giant viruses infecting unicellular algae [42]. This fact [42] suggests the existence of a mechanism that works during the horizontal transfer of genes between giant viruses in protozoa and their “hosts”, including between the megavirus *Mollivirus sibericum* and its “host”. The replication of this virus begins with its entry to the amoeba *A. castellanii* by phagocytosis. The virus then gets into the nucleus of the protozoon to start replicating its DNA on the edge of the deformed nucleus, in a “viral particle factory” [88]. There is evidence that, as a result of the infection of the amoebae by *Mollivirus sibericum*, as soon as 4–5 h of the infection, their number drops dramatically [88].

The last giant virus infecting protozoa and making up no family is the megavirus Kaumoebavirus, identified in 2016. It infects amoebae of the genus *Vermamoeba vermiformis* which live in waste water in the southern part of Jeddah, Saudi Arabia (Table 3). This virus has a icosahedral capsid with a diameter of 250 nm, and its replication starts as soon as about 3 h after the infection, that is, after its entry to the protozoon’s cell by phagocytosis as after that time, the viral particles arrange themselves into aggregates of 2–4 virions. After 6 h of infection, they are found near the amoebal nucleus, in the so-called “viral particle factory” [57]. The virus’ descendant particles are found after 16 h of infection of the amoebae, whereas their lysis occurs after 20 h [57]. The research [57] has also shown that the replication of Kaumoebavirus does not change the morphology of the amoebal nucleus. Unlike in other megaviruses infecting protozoa and like in APMaV, Golden *marseillevirus*, Cannes 8 and Faustovirus E12, the genome of Kaumoebavirus is circular dsDNA containing 350,731 base pairs and encoding 465 proteins, 59% of which have analogues among proteins of other giant viruses in protozoa, especially in viruses of the genus Faustovirus (43%) and in those of the family *Asfarviridae* (23%), infecting vertebrates (swine) [57]. According to Bajrai et al. [57] it is suggested that, although *Kaumoebavirus* is quite close related to faustoviruses and asfaviruses, it may in the future constitute a new genus of megaviruses which will then make up a new family of giant viruses [57].

### The role of giant viruses infecting protozoa in the natural environment

The role of giant viruses infecting protozoa (amoebae, flagellates) is very important from the biological point of view because in aquatic environments, both amoebae and flagellates are the main components of the zooplankton, whereas the megaviruses that are “hosts” to virophages (Tables 1, 2, 3) have an impact on the protozoal population, thus being zooplankton regulators. There is evidence that the number of protozoa decreases after they are infected. However, the size of their population is conditioned by the presence of virophages in the megaviruses [6–9, 31]. It has been reported that after the infection of amoebae and flagellates by giant viruses and virophages, the number of the protozoa drops but the drop is more drastic when the protozoa are infected by megaviruses only. This supports the fact that giant viruses have an impact on the sizes of the populations of amoebae and flagellates in aquatic environments [6–9, 31]. However, when it comes to the infection of protozoa by virophages of giant viruses, the mortality rates for amoebae and flagellates decrease because the virophages diminish the adverse impact of the megaviruses [9]. This observation was very evident in the case of the Zamilon virophage, infecting the giant virus Mont1 which colonises the amoebae *Acanthamoeba polyphaga* [3]. However, it has also been shown that the damaging impact of Zamilon on the megavirus Mont1 is partly eliminated by the MIMIVIRE system which is present in Mont1 [3] and which has also been reported to be present in other giant viruses, e.g. in the megavirus *Pithovirus sibericum* [35] as well as in ACMV and CroV of the family *Mimiviridae* [5, 6]. This system is similar to the CRISP-Cas mechanism which is very common among bacteria and archaea [9] and which is based, e.g. in the megavirus Mont1, on the repeats of groups of short palindrome sequences that form after RNA transcription and serve as a guide to enzymatic proteins (including helicases and nucleases) that are responsible for degradation of foreign nucleic acids. This possibility of the foreign DNA being digested by such short palindrome sequences is included in between the repeated sequences and during further infections; these sequences become immediately active, e.g. in bacteria and archaea, in response to the foreign DNA, that is the DNA of bacteriophages and in the case of giant viruses—the DNA of virophages. Research into the MIMVIRE system in the giant virus Mont1 has shown that it has as many as 28 nucleotide repeats of palindrome sequences that do not contain ORFs [48]. It has also been demonstrated [9] that, irrespective of the existence of the MIMIVIRE defence mechanism in Mont1 against the Zamilon virophage, its impact on the number of the giant viruses Mont1 is apparent [9]. The latter fact and the reported damaging impact of virophages on giant viruses living in protozoa show that virophages protect the protozoa being their “hosts”, hence they are called “friends” of protozoa [89]. It has been demonstrated, for example in the case of the giant virus ACMV infected by the Sputnik virophage which replicates in the amoeba *A. polyphaga* that such a state reduces amoebal lysis by 13% compared to the number of amoebae infected by ACMV only [5, 6]. A similar picture has been observed in a culture of the flagellate *Cafeteria roenbergensis* infected by the giant virus CroV and the virophage Mavirus [5]. Research results have also proven that megaviruses also take part in the microbial loop as they strongly encourage the growth of unicellular algae, corals and bacteria in, for example, Antarctic lakes [27]. It has also been shown that virophages also take part in the microbial loop, also in relation to bacteria as there is evidence that superinfection of protozoa by a giant virus and BABL1 bacteria results in an increased population of BABL1 bacteria but only when the megaviruses have virophages, the presence of which contributes to the reduction in their number and, as a result, to an increase in the number of BABL1 bacteria due to the competition between the bacteria and the giant viruses for the host (protozoa), as by decreasing the number of giant viruses, the virophages increase the quantity of food (protozoa) for the bacteria [27].

## Summary

Since the first reports on giant viruses in protozoa, published in 2003, they have been described in a growing number of reports. There are currently two families of these viruses (*Mimiviridae* and *Marseilleviridae*) (Tables 1, 2), a group of viruses making up no genera, four viral species (*Pandoravirus, Pithovirus, Faustovirus, Mollivirus)* and one genus, *Kaumoebavirus*, which makes up no family (Table 3). These megaviruses infecting amoeba and flagellates as well as invertebrate and vertebrate animals, including mammals (humans, rodents and animals) have many features that distinguish them from the hitherto described viruses, including giant viruses, inter alia in respect of the occurrence of elements identified in Archaea or Eukarya, which inter alia has formed the basis for classifying them into the new group of factors standing out from the three domain classification (TRUC) and for which a separate taxonomic unit has been proposed: Megavirales [2, 4, 12, 13]. Data about giant viruses in protozoa, in particular data on their “structure”, are also very important in the context of the evolution of viruses [86, 87], which also may form the basis for their division [4, 90]. Moreover, the identification of virophages in giant viruses infecting protozoa that are “elements” which have not been previously identified and which affect and condition the quantity of zooplankton in aquatic environments is an important fact for a practical reason in natural sciences. Plus, the identification of megaviruses in the human–environment (water, soil), but especially in the human and animal (sheep, cattle, rodents) excrement, blood and tissue, including, as a component of the microbiome of mammals (humans, animals) becomes an important fact in the research into health and diseases of vertebrates, including humans and livestock [20, 23, 24, 26, 31, 62, 66, 78]. It has also been demonstrated [24] that the infection of a human by the megavirus APMV opens up a new, hitherto unknown way for immune response in the fight against germs, including viruses.
